# Overweight and constipation in adolescents

**DOI:** 10.1186/1471-230X-11-40

**Published:** 2011-04-17

**Authors:** Mariana L Costa, Julyanne N Oliveira, Soraia Tahan, Mauro B Morais

**Affiliations:** 1 Division of Pediatric Gastroenterology, Escola Paulista de Medicina, Federal University of São Paulo, São Paulo, Brazil

**Keywords:** constipation, overweight, adolescent health

## Abstract

**Background:**

The association between overweight and gastrointestinal symptoms has been recently studied in the literature; however, few studies have evaluated the association between overweight and constipation in adolescents in a community-based sample. The aim of this study was to analyze the prevalence of constipation and its association with being overweight in a community-based survey with adolescents.

**Methods:**

This cross-sectional study included 1,077 adolescents who were enrolled in five schools in the city of Sao José dos Campos, Brazil. Constipation was defined according to modified and combined Rome III criteria for adolescents and adults. Being overweight was defined as a body mass index (BMI) that was equal to or greater than that of the 85^th ^percentile for age and gender.

**Results:**

Constipation was diagnosed in 18.2% (196/1077) of the included adolescents. There was no significant difference in the prevalence of constipation in males and females who were both younger and older than 14 years. Fecal incontinence was observed in 25 adolescents, 22 (88.0%) of whom were diagnosed as being constipated. The prevalence of being overweight was found in 13.5% (145/1077) of the study population. The prevalence of constipation was observed to be similar in adolescents who were (19.4%; 28/144) and were not (18.0%; 168/933) overweight (p = 0.764; OR = 1.10). Fecal incontinence that was associated with constipation was more frequent in adolescents who were overweight (37.0%; 8/28) than in adolescents who were not overweight (8.5%; 14/168; p = 0.005; OR = 4.40).

**Conclusions:**

The prevalence of constipation was high among the investigated adolescents. There was no association between being overweight and constipation; however, an association between being overweight and fecal incontinence in constipated adolescents was confirmed.

## Background

The prevalence of being overweight and obesity has been increasing worldwide [1], and it has been verified that overweight adolescents are at risk for disease in adulthood because this condition tends to be maintained and is related to secondary conditions, such as type 2 diabetes mellitus, dyslipidemia, arterial hypertension and osteoarthritis [1].

Functional gastrointestinal diseases are also prevalent in infancy, adolescence and adulthood [2-4]. A recent controlled study identified an association between functional gastrointestinal diseases in pediatric patients and overweight and obesity [5]. Functional constipation and fecal incontinence were associated with overweight or obesity in children and adolescents who attended specialized pediatric gastroenterology and endocrinology clinics [5-8].

In 1981, the evaluation of a sample of 1987 subjects who were aged between 6 and 70 years living in Italy showed that constipation was more frequent in individuals with obesity [9]. Other population-based studies of American adults did not find an association between constipation and overweight [10,11]. On the other hand, a community-based study that evaluated adults in Tehran, Iran found an association between fecal incontinence and higher values of body mass index [12]. A high prevalence of overweight and obesity in Iranians with functional constipation was also observed [12].

Adolescence is considered to be a risk period for the adoption of inadequate feeding habits that may constitute a risk for constipation and overweight. Studies evaluating the association of functional gastrointestinal disorders and obesity that have included the adolescent age group were performed in specialized centers in the United States of America [5-8]. Therefore, to our knowledge, there has been only one community-based study of adolescents, which found an association between low dietary fiber intake and overweight but did not link constipation to overweight [13].

The importance of studying the relationship between functional gastrointestinal symptoms and obesity has been recently highlighted [14]. Considering that few articles have evaluated the intestinal habits of adolescents and their association with overweight, the aim of this study was to analyze the prevalence of constipation with and without fecal incontinence and its association with overweight in a community-based survey of adolescents.

## Methods

This cross-sectional study involved adolescents who were aged between 10 and 18 years and included five public or private schools in the city of Sao José dos Campos. São José dos Campos is located 91 kilometers away from the city of São Paulo that is the capital of the State of São Paulo in the southeast region of Brazil. It has a population of approximately 615,000 inhabitants, of whom more than 95% live in the urban area. After obtaining permission to carry out the project from the directors of each school, the research project was detailed to pedagogic supervisors or teachers of these institutions. These people were responsible for both the delivery of the sealed envelopes that contained the questionnaires, the free consent forms for parents/guardians and for any clarification for each student in the classroom, both for guidance in completing the questionnaire and ensuring the signing of the consent form by those responsible. Consequently, there was no contact between the researchers and the students and/or those responsible for them. Of the 2,500 questionnaires that had been issued, 1282 (51.3%) were returned. Of these, 205 (15.9%) were excluded because of incomplete or inadequate completion. Therefore, the sample that was considered in this study was composed of 1,077 adolescents.

The questionnaire contained multiple-choice questions that had been formulated in simple language, were easy to comprehend and had been validated in a prior pilot study of adolescents of the same age group. The questions covered the following characteristics of intestinal habits: frequency of defecation during the week, history of painful or hard bowel movements, characteristics of the stools (consistency, format and size that may obstruct the toilet), sensation of incomplete evacuation, history of fecal incontinence and abdominal pain that may or may not improve after defecation.

Constipation was defined according to the modified and combined Rome III criteria for adolescents [3] and adults [4], taking into account the possible answers to the administered questionnaire. Therefore, the adopted diagnostic criteria for constipation included two or more of the following: (1) two or fewer defecations in the toilet per week, (2) a history of painful or hard bowel movements, (3) hard stools that resembled a sausage but have cracks on their surface or separate hard lumps, (4) a sensation of incomplete evacuation, (5) a history of large diameter stools that may obstruct the toilet and (6) a history of fecal incontinence. For characteristics 1 to 4, the questions referred to the predominant defecation pattern that occurred more than 25% of the time. There was also a question concerning the occurrence of abdominal pain ("always" or "almost always") and another question addressing whether this abdominal pain improves with defecation as is observed in irritable bowel syndrome [3,4].

The investigated adolescents also provided the following information: age (in years), date of birth, gender, weight (in kilograms) and height (in meters). Previous studies [5,15-17] of adolescents found a strong correlation between reported and measured values for weight and height, showing that the referred information may be used as an indication of nutritional status. This data were used for calculation of the body mass index (BMI = body weight divided by the square of the height). The BMI was interpreted with consideration of the age and gender of the subject. BMI is internationally recognized as a metric for diagnosing the nutritional status of individuals during adolescence [18]. As reference standards, data from the Center for Disease Control and Prevention (2000) were used [19]. Overweight was defined when the BMI was equal to or higher than that of the 85^th ^percentile [18]. Adolescents with a BMI that was below that of the 85th percentile were considered to not be overweight.

The studied population was divided into two groups according to age: under 14 years and 14 years of age or older. Continuous variables that did not have a normal distribution were expressed by median and the 25^th ^and 75^th ^percentiles and were compared using the Mann-Whitney test. Continuous variables with normal distributions were expressed by means and standard deviations and were compared via Student's t test. Mann-Whitney and Student's t tests were performed using SigmaStat Software (Systat Software, Inc., San Jose, California, United States of America). For categorical variables, the Chi-squared (χ^2^) test or Fisher's exact test was used. The magnitude of the association between variables was estimated using odds ratios (OR) with a confidence interval (CI) of 95.0%, which was calculated using EPI-INFO software (Centers for Disease Control and Prevention, Atlanta, Georgia, United States of America). The alpha error was fixed at 5%. This study was approved by the Research Ethics Committee of the Federal University of Sao Paulo (number 1545/03).

## Results

A total of 1,077 questionnaires were analyzed, of which 46.0% (495) had been completed by male adolescents and 54.0% (582) by female adolescents. Constipation was diagnosed in 18.2% (196/1077) of the adolescents. Table 1 depicts the occurrence of constipation according to age and gender. There was no significant difference in the prevalence of constipation in males and females who were both younger and older than 14 years of age. On the other hand, constipation was more prevalent in females who were older than 14 years (22.6%) in comparison to females who were younger than 14 years of age (15.2%; p = 0.03). The question relating to the occurrence of abdominal pain as "always" or "almost always" was positively answered by 196 (18.2%) out of the total sample of 1077 adolescents. Abdominal pain in constipated adolescents (35.7%; 70/196) was more frequent (p < 0.0001) than in non-constipated individuals (8.8%; 78/881). Abdominal pain was more frequent in females who were older than 14 years of age in comparison to males of the same age group (59/319; 18.5% and 31/274; 11.3%, respectively, p = 0.021). In test subjects who were younger than 14 years of age, these values were 12.1% (32/263) in females and 11.8% (26/221; p = 0.996) in males. Abdominal pain improvement after defecation was indicated by 70.0% (49/70) of constipated adolescents with abdominal pain and 64.1% (50/78) of the non-constipated adolescents with abdominal pain (p = 0.558).

**Table 1:** Constipation in adolescents from São José dos Campos, Brazil, according to age and gender

	**No**^**1**^	**Yes**^**2**^	**% Yes**^**2**^	p	OR
**< 14 years**					
Male	187	34	15.4	0.941*	1.01 (0.60; 1.71)^‡^
Female	223	40	15,2		
Total	410	74	15.3		
**≥ 14 years**					
Male	224	50	18,2	0.232*	0.77 (0.50-1.17)^‡^
Female	247	72	22,6		
Total	471	122	20,6		

1. Without constipation,

2. With constipation

*Chi-squared test; ^‡^Odds Ratio (95% confidence interval).

Chi-squared test for age < 14 years versus ≥ 14 years: Male (p = 0.469); Female (p = 0.033).

Fecal incontinence was observed in 25 adolescents, 22 (88.0%) of whom were diagnosed as constipated. The other 3 adolescents who were aged between 10.2 and 10.3 years with fecal incontinence did not present any other criteria for the diagnosis of constipation and were classified as having normal intestinal habits. Constipated adolescents with fecal incontinence were aged between 10.8 and 18.1 years. The mean age (14.2 ± 2.3 years) of these 22 constipated adolescents with fecal incontinence was significantly higher than the mean age of the 3 adolescents with fecal incontinence without constipation (11.0 ± 0.6 years; p = 0.029). Therefore, the overall frequency of fecal incontinence in the studied population was 2.3% (25/1077; 11 males and 14 females).

The prevalence of overweight was 13.5% (145/1077). Table 2 shows that the prevalence was higher in adolescents who were younger than 14 years in both genders. In adolescents who were younger than 14 years of age, overweight was more prevalent in males.

**Table 2:** Overweight in adolescents from São José dos Campos, Brazil, according to age and gender

	**No**^**1**^	**Yes**^**2**^	**% Yes**^**2**^	p	OR
**< 14 years**					
Male	168	53	24.0	0.020*	1.76 (1.09-2.85)^‡^
Female	223	40	14.7		
**≥ 14 years**					
Male	248	26	9.5	0.667*	1.18 (0.64-2.17)^‡^
Female	293	26	8.2		

1. Without overweight; 2 With overweight

* Chi-squared test; ^‡^Odds Ratio (95% confidence interval).

Chi-squared test for age < 14 years versus ≥ 14 years: Male (p = 0.000); Female (p = 0.011).

Table 3 shows that there was no association between overweight and constipation in this community-based sample. There was no statistically significant difference between the BMI of the adolescents who had (median = 19.4 and 25^th ^and 75^th ^percentiles of 17.6 and 21.4 Kg/m^2^, respectively) and did not have (median = 19.3 and 25^th ^and 75^th ^percentiles of 17.6 and 21.6 Kg/m^2^, respectively) constipation (p = 0.941, as determined by the Mann-Whitney test). In the constipation group, the 22 adolescents who had fecal incontinence presented higher (p = 0.023) BMI values (median = 21.1 and 25^th ^and 75^th ^percentiles of 18.7 and 23.7 Kg/m^2^, respectively) in comparison to the 174 adolescents who had constipation but without fecal incontinence (median = 19.2 and 25^th ^and 75^th ^percentiles of 17.4 and 21.5 Kg/m^2^, respectively).

**Table 3:** The association between constipation and overweight in adolescents from São José dos Campos, Brazil

		**No**^**1**^	**Yes^2^**	**% Yes**^**2**^	p	OR
**Overweight**	**Yes**	116	28	19.4%	0.764*	1.10 (0.69-1.75)^†^
	**No**	765	168	18,0%		

1. Without constipation, 2. With constipation

* Chi-squared test; ^†^Odds Ratio (95% confidence interval).

The frequencies of abdominal pain in adolescents with and without overweight were similar [11.1% (16/144) and 13.3% (124/933), respectively; p = 0.555].

The association between fecal incontinence and overweight in adolescents with constipation is shown in Table 4. Fecal incontinence was associated with overweight (odds ratio = 4.40, 95% confidence limits: 1.47 and 13.05).

**Table 4:** The association between fecal incontinence and overweight in adolescents with constipation from São José dos Campos, Brazil

		**No**^**1**^	**Yes**^**2**^	**% Yes**^**2**^	P	OR
**Overweight**	**Yes**	20	8	37.0	0.005*	4.40 (1.47; 13.05)^†^
	**No**	154	14	8.5		

1. Without fecal incontinence associated to constipation; 2. With fecal incontinence associated to constipation

* Fisher exact; ^†^Odds Ratio (95% confidence interval).

## Discussion

The prevalence of functional constipation was 18.2% and was more prevalent in females who were older than 14 years of age than it was in female adolescents who were younger than 14 years of age. Despite the adoption of different criteria for diagnosing functional constipation, the prevalence was similar to another study of adolescents in São Paulo [13], which found constipation more frequently (but without statistical significance) in female teenagers (25.2%) than in males (16.9%) in a population of private school students. In students from public schools of São Paulo, these values were 31.6% and 19.6%, respectively (p = 0.051) [13]. The data obtained in São José dos Campos showed that the prevalence of constipation in females was higher after 14 years of age, which demonstrates the moment in adolescence that the increased prevalence of constipation in females begins; however, this increased prevalence was not sufficient to provide a significant difference in relation to male adolescents of the same age group. These data contrast those observed in specialized pediatric clinics, wherein severe constipation was observed to be more prevalent in the male gender [3,20]. Another interesting result is the observed higher occurrence of abdominal pain in adolescents with constipation (35.7%) in comparison to those without constipation (8.8%). This symptom could be related to irritable bowel syndrome, especially if the pain disappears after defecation. It could be speculated that some of the adolescents who were diagnosed as having functional constipation in our study may suffer from irritable bowel syndrome; however, the questionnaire used in this study did not allow for the recognition of irritable bowel syndrome. In the non-constipated adolescents, abdominal pain occurred in 8.8% of the population. This value is lower than that observed in students attending Chicago public schools who participated in a prospective cohort study that found the overall prevalence of abdominal pain to be 38% and that of constipation to be 8% [21]. The observed difference may be explained by differences in the questionnaires, strategies of data collection and focuses of the studies. On the other hand, our result concerning the occurrence of abdominal pain agrees with similar cross-sectional, community-based studies [22,23].

At specialized pediatric gastroenterology clinics, fecal incontinence is frequently observed and may be the reason for referral to the specialist. On the other hand, few studies have evaluated the occurrence of fecal incontinence in the general population. A British study involving children and adolescents at primary schools who were aged between 5 and 6 years and 11 and 12 years found fecal incontinence in 4.1% and 1.6% of the children, respectively [24]. In the present study, fecal incontinence was observed in 2.3% (25/1077) of the total cohort of adolescents and was more frequent in adolescents with constipation. This result is similar to the prevalence (1.7%) of fecal incontinence in adults according to a cross-sectional, community-based study in Iran [12].

The prevalence of overweight was 13.6% in the adolescents who were evaluated in this study. These data agree with the prevalence of overweight in a national study that carried out in Brazil, which identified overweight in 12.3% of adolescents [25]. There was also agreement with the literature in relation to the observed higher frequency of overweight in male adolescents who were [26,27] younger than 14 years of age [25].

The primary aim of this study was to evaluate the association between overweight and constipation. There was no association between overweight and constipation (Table 3). These data agree with other community-based studies of adolescents and adults [10,11]. On the other hand, the collected data disagree with studies that have been conducted in specialized pediatric gastroenterology clinics [5-8]. Moreover, in our study, fecal incontinence in constipated patients was associated with overweight (Table 4). This result agrees with studies involving adults and children and adolescents who have been studied in specialized centers [5-8]. The previous community-based study involving adolescents did not evaluate the relationship between overweight and fecal incontinence [13]. Taking into account that fecal incontinence appears in more severe cases of constipation, it may be hypothesized that the association between constipation and overweight is more evident in patients with severe gastrointestinal motility disorders.

In this context, it was suggested that overweight and severe functional gastrointestinal disorders may be linked through a chronic inflammatory status in which the liberation of proinflammatory cytokines may be present in both conditions [12,28]. Epidemiological data indicate that obesity is associated with a wide range of gastrointestinal symptoms that are secondary to functional gastrointestinal disorders, such as irritable bowel syndrome and dyspepsia [10,11,29].

Another question that may be raised is that obese patients may have more problems cleaning their anus after defecation. In our study, the highest BMI in adolescents with incontinence was 29.3 Kg/m^2 ^(data not shown), which is not consistent with morbid obesity. Therefore, it is improbable that this degree of overweight may be associated with a physical difficulty in maintenance of hygiene following defecation. Additionally, our results show a strong association between fecal incontinence and constipation, suggesting that this finding should be interpreted in the scenario of functional gastrointestinal disorders.

## Conclusion

In conclusion, the present study found an elevated prevalence of constipation and overweight in adolescents. An association between constipation and overweight was not observed, although fecal incontinence was more prevalent in constipated adolescents who were overweight.

## Competing interests

The authors declare that they have no competing interests.

## Authors' contributions

MLC: analysis and interpretation of data; statistical analysis, drafting of the manuscript; final approval of the version to be published; JNO: study concept and design; acquisition of data; drafting of the manuscript; statistical analysis; final approval of the version to be published; ST: study concept and design; analysis and interpretation of data; drafting of the manuscript; statistical analysis; study supervision, final approval of the version to be published; MBM: study concept and design; analysis and interpretation of data; drafting of the manuscript; statistical analysis; study supervision, final approval of the version to be published.

## Pre-publication history

The pre-publication history for this paper can be accessed here:

http://www.biomedcentral.com/1471-230X/11/40/prepub
